# Plica Neuropathica (Polonica) Secondary to Diffuse Alopecia: A Case Report and Literature Review

**DOI:** 10.7759/cureus.93402

**Published:** 2025-09-28

**Authors:** Alondra Saray Polanco-Llanes, Abraham Isaí Cabello-Hernández, Paulina Nundehui Cortés-López, Ximena Gintare Alvarez-Estrada, Genaro Briseño-Gascón

**Affiliations:** 1 Department of Dermatology, Hospital General "Dr. Manuel Gea González", Mexico City, MEX; 2 Department of General Medicine, Instituto Politécnico Nacional, Mexico City, MEX; 3 Department of Dermatopathology, Hospital General "Dr. Manuel Gea González", Mexico City, MEX

**Keywords:** alopecia, androgenetic alopecia, diffuse alopecia, nonscarring alopecia, plica neuropathica, plica polonica

## Abstract

This article aimed to report a case of plica neuropathica (polonica) associated with diffuse alopecia, highlighting its diagnostic challenges and key distinguishing features. A 62-year-old female patient with a history of hospitalization presented to our department with a one-month history of increased hair loss and intermittent itching of the scalp. Upon examination, she presented with decreased hair density, pseudoalopecic areas, and the presence of plica neuropathica. Trichoscopy showed vellus hairs, anisotrichosis, yellow dots, and hairs with Pohl-Pinkus constrictions. Based on the percentages of anagen and telogen hairs in the presence of miniaturization in the histopathological study, a diagnosis of androgenetic alopecia with acute telogen effluvium was made. Treatment with topical minoxidil was initiated. The patient is being followed up by our service. Plica neuropathica, also known as plica polonica, is a rare condition characterized by irreversible matting and twisting of hair, resulting in a compact, keratinized, and water-resistant mass. It has been associated with the use of hair care products such as shampoos containing cationic surfactants, certain medications, inadequate hair hygiene, or excessive friction. In the literature, it has been described in association with alopecia areata and trichotillomania, but not with diffuse alopecia. Plica polonica is an infrequent clinical finding, and its occurrence in the context of diffuse alopecia has not been previously documented. This subtype of alopecia is challenging due to the lack of specific clinical signs. This case highlights the importance of considering other causes of alopecia associated with plica neuropathica.

## Introduction

Plica neuropathica, or plica polonica, is a rare condition characterized by irreversible twisting and tangling of the hair, resulting in a hard, impermeable mass of keratin. It is known to be multifactorial. Factors such as religious practices, the use of shampoos containing cationic surfactants, and excessive rubbing of the hair shafts, which generate electrostatic attraction, contribute to the formation of knots and tangles. On the other hand, impaired self-care (such as that observed in psychiatric disorders, prolonged hospitalizations, or influenced by personal beliefs) constitutes an additional risk factor for its onset [1]. Likewise, contact dermatitis, the use of drugs, and infections such as pediculosis and ringworm of the head have been associated with it [2,3].

## Case presentation

**Figure 1:**
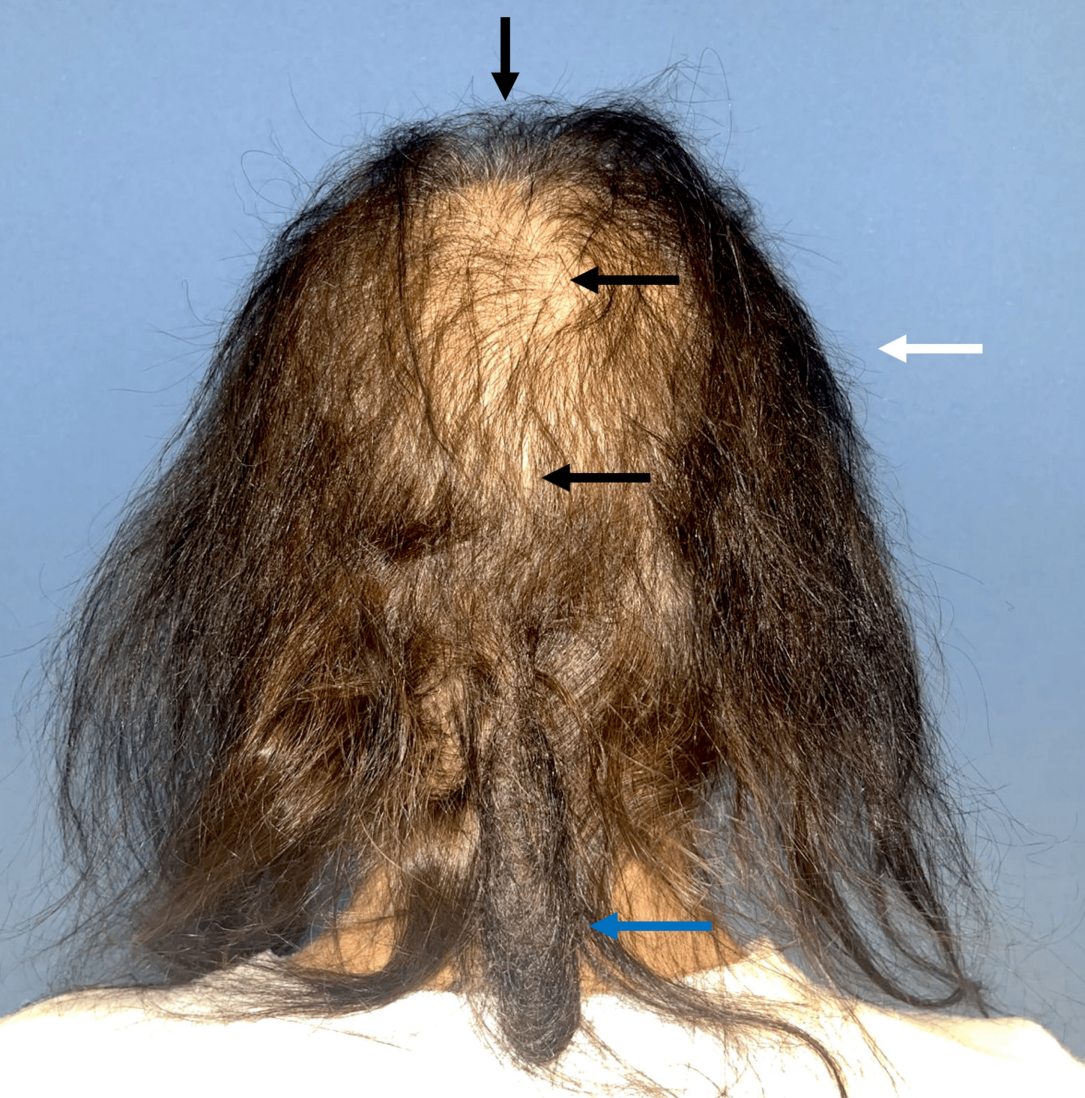
Clinical image of the scalp Decreased hair density (white arrow), pseudoalopecic areas on the vertex, occipital region, and temporal areas (black arrow); presence of a compact mass of dry, dull, tangled hair in the occipital region (plica neuropathica, blue arrow)

A 62-year-old female patient with a history of type 2 diabetes and a one-month hospital stay due to complicated emphysematous cystitis presented to our service with a one-month history of increased hair loss and intermittent itching of the scalp. During hospitalization, she noticed increased hair loss, which had begun discreetly in the previous months. The patient reported a history of portal vein thrombosis and acute myocardial infarction, as well as use of metformin, clopidogrel, and apixaban. On examination, she presented with a localized dermatosis on the head affecting the scalp in the vertex, temporal, and occipital regions, characterized by decreased hair density, pseudoalopecic areas, and the presence of plica polonica in the occipital area (Figure 1).

**Figure 2:**
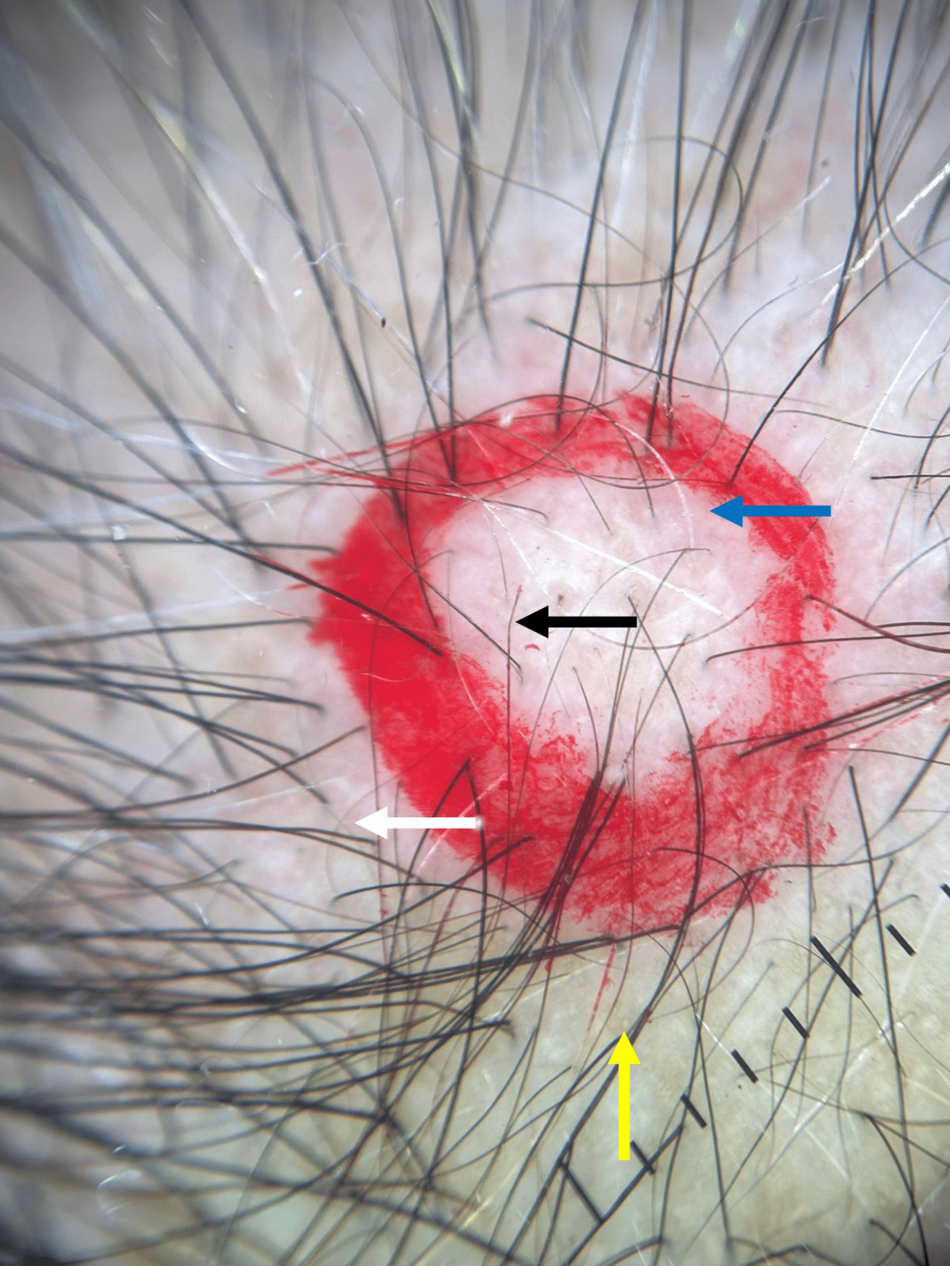
Trichoscopic findings on the scalp Vellus hairs (white arrow), yellow dots (yellow arrow), anisotrichosis (blue arrow), and hairs with Pohl-Pinkus constrictions (black arrow)

**Figure 3:**
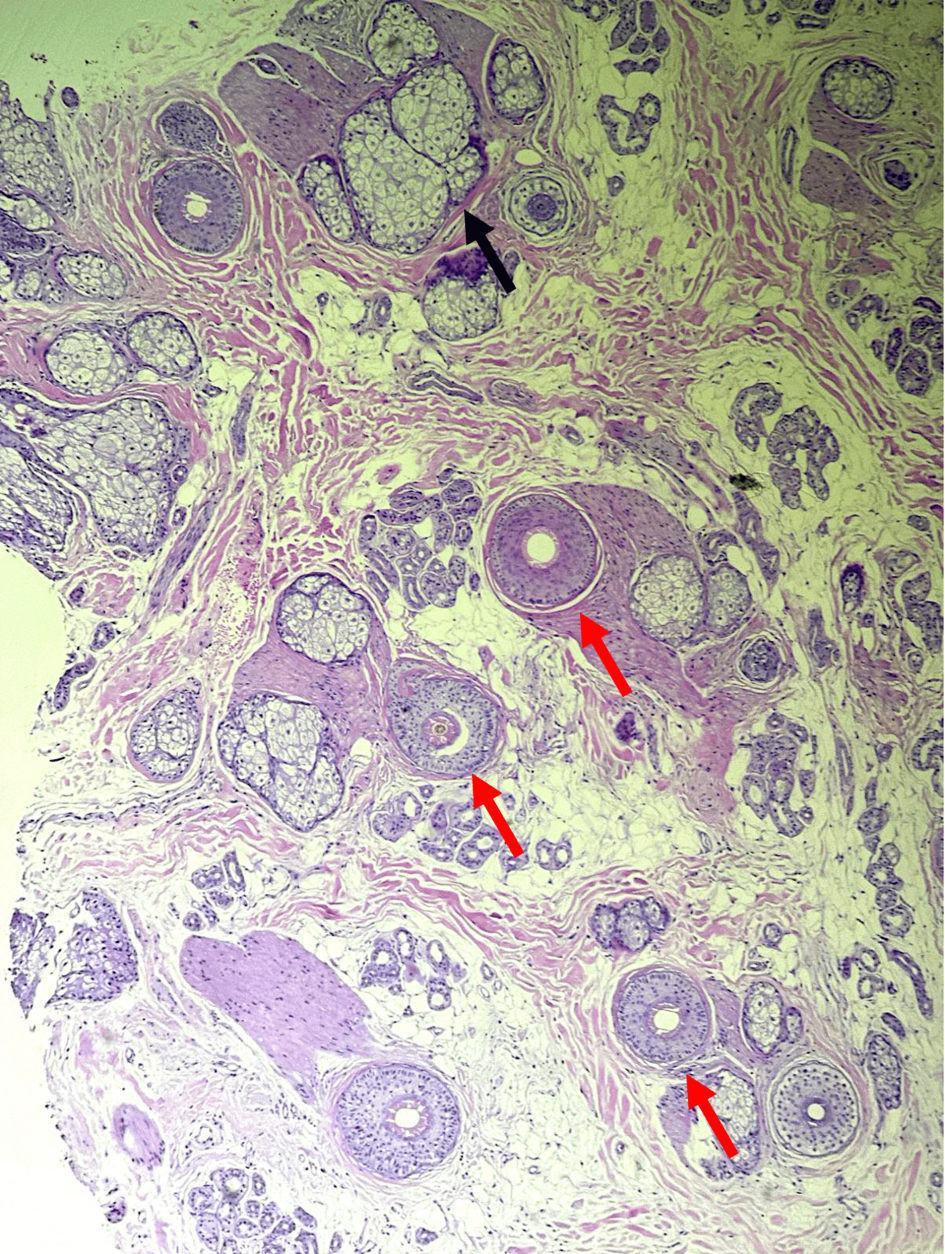
Histological image of the scalp The terminal (T) to vellus (V) ratio is 3.2:1 (red arrow) with no pigment loss or dystrophic shafts. Follicular stumps without inflammatory infiltrate and sebaceous glands without alterations (black arrow) (hematoxylin-eosin stain; original magnification ×10)

Trichoscopy showed vellus hairs, anisotrichosis, yellow dots, and Pohl-Pinkus constrictions (Figure 2). Laboratory studies revealed 25-OH vitamin D deficiency (21.71 ng/mL) and mild anemia (Hb 13.28 g/dL) (Table 1). Histopathological examination revealed a homogeneous distribution of follicular units, with a total of 25 hair follicles, 19 terminal and six vellus (terminal ratio: 3.2:1), 76% of follicles in anagen, and 24% in catagen/telogen, without pigment loss or dystrophic shafts, follicular stumps without inflammatory infiltrate, and sebaceous glands without alterations (Figure 3). Based on the percentages of anagen and telogen hairs in the presence of miniaturization, the diagnosis of androgenetic alopecia with acute telogen effluvium was made. The patient was treated with minoxidil 5% spray once daily. She was also advised to cut her matted hair. The patient is being followed up by our service.

**Table 1:** Patient's laboratory results MCV: mean corpuscular volume; BUN: blood urea nitrogen; TSH: thyroid-stimulating hormone; T4: thyroxine; 25-(OH) vitamin D: 25-hydroxy vitamin D

Laboratory parameters	Patient’s results	Normal values
Leukocytes (cells/µL)	8.8	4.5-11.0
Neutrophils (cells/µL)	5.4	1.5-8
Lymphocytes (cells/µL)	2.3	1-4.8
Hemoglobin (g/dL)	13.2	12-15
Hematocrit (%)	39	36-48
Transferrin (mg/dL)	217	245-370
Ferritin (ng/mL)	379	24-336
MCV (fL)	98.5	80-100
Platelet (10^3^/µL)	209	150-450
Glucose (mg/dL)	98	<100
BUN (mg/dL)	18	6-20
Urea (mg/dL)	39	17-43
Creatinine (mg/dL)	0.7	0.6-1.3
TSH (µUI/mL)	5.7	0.4-4
T4 (ng/dL)	0.79	5-11
25-(OH) vitamin D (nmol/L)	21.71	30-60

## Discussion

Androgenetic alopecia is the most common type of nonscarring alopecia, with a genetic predisposition present in 80% of cases. It is associated with increased sensitivity to androgens and a reduction in the anagen phase of the follicular cycle. Clinically, in women, it presents as a diffuse decrease in hair density on the crown and upper parietal region [4]. Trichoscopic diagnosis shows anisotrichosis, yellow and white dots, honeycomb pigmentation, focal atrichia, and brown peripilar sign [5].

Telogen effluvium is characterized by diffuse nonscarring hair loss. There is a premature transition of hair follicles from the anagen phase to the telogen phase. It is caused by drugs such as retinoids, antithyroid drugs, anticonvulsants, anticoagulants, antidepressants, etc. In terms of nutritional deficiencies, it has been associated with deficiencies in zinc, iron, riboflavin, and vitamin D, as well as general malnutrition. Physical or emotional stress is also a frequent trigger, such as severe illnesses, hemorrhages, trauma, or major surgery [6-8]. In trichoscopy, there are no pathognomonic findings; however, the presence of regrowing hairs and follicles with a single emerging shaft may be suggestive in the absence of specific signs of other alopecias [5].

Plica neuropathica, also known as plica polonica or trichoma, is a rare acquired hair disorder characterized by the formation of a compact mass of tangled, moist, foul-smelling, and sticky hair composed of irreversibly tangled braids [9]. During the 19th century, it was a common condition in Poland, related to pediculosis and poor hygiene practices [9]. The term neuropathic plica was first used by Le Page in 1884 [10] to describe a sudden case of tangled hair in a 17-year-old patient suffering from hysteria. It has previously been associated with trichotillomania and alopecia areata. It has been associated with contact dermatitis due to the use of dye with concomitant use of calcium acetate and aluminum sulfate powders [2]. However, its appearance in the context of diffuse alopecia has not been documented.

The mechanism of hair tangling is not fully understood; however, it is postulated that it is related to focal damage to the hair shaft cuticle, which exposes the underlying cortex with adhesive properties, promoting the agglutination of other hair shafts [11]. Three etiopathogenic groups have been described: physical conditions, chemical agents, and behavior [12]. Physical factors such as fine hair, hair density, and elasticity have been implicated in the pathogenesis [13]. The mechanical factors include a physical phenomenon known as felting, similar to that which occurs in the textile industry, where adjacent hair fibers are compacted due to friction and compression in a liquid medium. Additionally, the electrostatic attraction between the hair shafts promotes their adhesion. Finally, a fusion of viscous fluids occurs with the subsequent formation of lipotropic crystalline phases, a mechanism that is often triggered after the use of shampoos containing cationic surfactants [12]. 

The development of neuropathic plica secondary to drugs, mainly chemotherapeutic agents such as methotrexate, paclitaxel, and carboplatin, has been reported. In two cases related to azathioprine-induced pancytopenia, concomitant telogen effluvium and plica formation were documented, suggesting that the cuticular damage caused by these drugs may promote hair tangling [14,15]. It has also been reported to be associated with psychiatric disorders such as depression, schizophrenia, and autism related to repetitive hair manipulation [16,17]. Other etiologies include pediculosis, scabies, poor hygiene, ringworm of the scalp, use of natural dyes, infections, religious clothing, seborrheic dermatitis, and psoriasis rupioides [11,18,19].

In our patient, multiple predisposing factors coexisted, such as acute hair loss associated with systemic deterioration, nutritional deficiencies, loss of hair care during prolonged hospitalization, and possible damage induced by medications. All of these factors may have contributed to the development of the plica and possibly accelerated the progression of alopecia. This reinforces the hypothesis that structural damage to the hair shaft due to friction and alterations in the hair cycle due to diffuse alopecia may predispose to the formation of plica, even in the absence of obvious dermatological, psychiatric, or infectious conditions.

Treatment includes cutting the affected portion of hair and treating the predisposing factors [12]. In early cases, manual separation using organic solvents may be attempted [20]. This condition can be prevented by improving hygiene conditions, cutting hair regularly, applying oil, and combing it gently [20].

## Conclusions

Plica neuropathica (polonica) is an unusual hair disorder, and its association with diffuse alopecia had not previously been reported in the literature. This case highlights the importance to consider uncommon features such as plica polonica in patients with diffuse alopecia, beyond more commonly diagnosed disorders such as trichotillomania or alopecia areata. It also suggests the possible involvement of the patient's general systemic condition in its pathogenesis. Confirmation by histopathological study is key to distinguishing between possible etiologies.
